# Biomimetic CuCoO_2_ nanosheets reinforced with self-assembling peptide nanofibers for tumor photothermal therapy

**DOI:** 10.1039/d4ra07435a

**Published:** 2024-12-11

**Authors:** Mingjin Xu, Youyin Xu, Chenxi Du, Guanghui Gu, Gang Wei

**Affiliations:** a Department of Radiation Oncology, The Affiliated Hospital of Qingdao University Qingdao 266700 PR China guguanghui@qdu.edu.cn; b College of Chemistry & Chemical Engineering, Qingdao University 266071 Qingdao PR China weigroup@qdu.edu.cn

## Abstract

The flexible design and unique physical and chemical properties of self-assembled peptides have shown great potential for applications in the fields of materials science, life science, and environmental science. Peptide nanofibers (PNFs), as a kind of bioactive nanomaterials, possess excellent biocompatibility, flexible designability, and multifaceted functionalizability. In this work, we design and describe PNFs that self-assembled by peptide molecules as carriers for bimetallic nanosheets (BMNS), leading to the development of hybrid nanomaterials, BMNS–PNFs, with unique properties. The BMNS–PNFs exhibit a photothermal conversion efficiency (PCE) of up to 31.57%, and can be used as a potential nanoplatform for photothermal therapy (PTT) of lung tumour cells. Through the results, it is shown that the PNFs can reduce the cytotoxicity of BMNS–PNFs and that BMNS–PNFs have excellent cancer cell killing effects, with photothermal killing rates of more than 95% and 90% for lung cancer cells HCC2279 and PC9, respectively. Finally, the comprehensive PTT performance of BMNS–PNFs is analysed by Ranking of Efficiency Performance (REP), and the REP value of BMNS–PNFs is calculated to be 0.741. The peptide sequences used to assemble into PNFs in this study are instructive for functional design and structural modulation of molecular self-assembly, and the constructed bimetallic–biomolecular hybrid materials provide a potential strategy for medical bioengineering.

## Abstract 2

The conjugation of CuCoO_2_ bimetallic nanosheets with self-assembling peptide nanofibers leads to enhanced tumor photothermal therapy.

## Graphical abstract

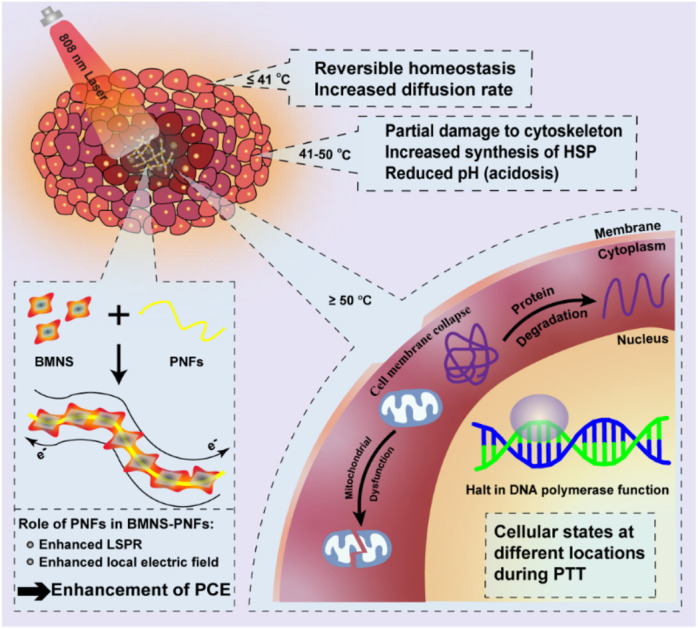

## 1. Introduction

Traditional cancer treatments, such as surgical resection, radiotherapy, and chemotherapy, often come with significant side effects, including toxic reactions,^1^ damage to the immune system,^2^ and tumor recurrence,^3^ all of which severely impact patients' quality of life. To overcome the adverse effects of conventional cancer therapies, more precise strategies have been developed that improve the efficacy of cancer treatment, including immunotherapy,^4^ targeted therapy,^5^ gene therapy,^6^ and photothermal therapy (PTT).^7^ PTT utilizes photothermal agents (PA) that can absorb near-infrared (NIR) light and convert it into thermal energy, rapidly generating localized high temperature to destroy the tumor cells.^8^ This non-invasive treatment method can, to some extent, avoid the risks and complications associated with surgery and minimize the damage to normal tissue cells.^9–11^ Furthermore, PTT is simple and effective in operation and can be combined with other treatment modalities to produce synergistic effects, thereby enhancing the overall therapeutic outcome.^12^ In the PTT process, the development of PAs with high photothermal conversion efficiency (PCE), low toxicity, and stability is crucial for advancing tumor therapies.^10,13^

Common PAs include carbon nanomaterials (such as graphene^14^ and carbon nanotubes^15^), organic small molecules,^16^ metal nanoparticles (NPs) (including Au NPs,^17^ Ag NPs^18^), as well as metal oxides or sulfides (such as TiO_2_,^19^ CuS^20^ and CuCoS^21^). Among them, metal oxides or sulfides have emerged as popular material candidates due to their high PCE, broad light absorption, excellent designability, and better economic feasibility.^22^ For example, CuCoS NPs synthesized by Zhu *et al.* have demonstrated outstanding PCE in the tumor PTT process.^21^ The formed CuCoS NPs catalyzed the production of reactive oxygen species (ROS) from H_2_O_2_ and deplete glutathione (GSH) in the tumor microenvironment, showing excellent tumor therapeutic effects. Additionally, metal oxides or sulfides possess abundant active sites, which can facilitate further modification and functionalization to endow enhanced functions.^23^ This includes enhancing the targeting ability of PAs, improving the effectiveness of PTT process, and increasing the biocompatibility of the photothermal materials.^24^ Although metallic nanomaterials have shown excellent therapeutic efficacy during PTT, some unfavourable effects still need to be considered. These include the biocompatibility, immune response, aggregation precipitation, biodegradability, and others. Therefore, metallic nanomaterials need to be modified to be better suited for tumor PTT.^25^ In another report, inspired by mussels, researchers used dopamine-conjugated hyaluronic acid to modify WO_3_ nanoparticles, in order to improve the biocompatibility of the hybrid material and enable tumor targeting.^26^ Further functional regulations of PAs can significantly enhance their clinical efficacy and safety.^27^

Peptides are biomolecules that formed by amino acids linked through amide bonds. By altering the amino acid sequence, length, and structure of peptides, self-assembling biomaterials with specific properties and functions can be constructed to meet various needs.^28–30^ Additionally, peptide molecules can further self-assemble through covalent or non-covalent bonds into nanomaterials with defined morphologies, such as nanospheres, nanofibers, nanoribbons, and nanosheets.^31,32^ Peptide nanofibers (PNFs) assembled from peptide molecules exhibited abundant modifiable sites, excellent biocompatibility, and controllable self-assembly process, as well as robust functionalization characteristics.^33^ Due to these advantages, PNFs have become a preferred choice for the biomimetic design and fabrication of various functional biomaterials.^34^ Particularly for the development of photothermal therapeutic materials, PNFs can enhance the biocompatibility of PAs and reduce the biotoxicity of metallic nanomaterials, which in turn enhances the overall effect of tumor PTT.^35^ In addition, targeted peptide self-assembled nanomaterials combined with PAs can also enhance the aggregation of PAs at the tumor site, which can enhance the photothermal killing effect on tumors.^31^

**Scheme 1:**
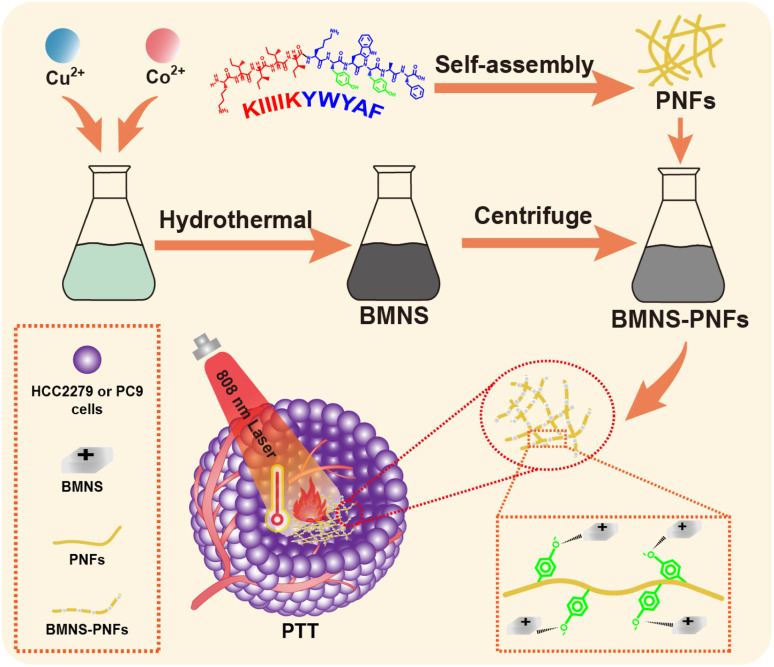
Schematic representation of the synthesis of BMNS–PNFs for PTT of cancer cells.

In this study, we design a self-assembling peptide with a sequence of KIIIIKYWYAF based on previous work,^36^ which successfully self-assembles into PNFs in an aqueous solution, providing an excellent carrier for PAs. As shown in Scheme 1, bimetallic nanosheets (BMNS, CuCoO_2_) with superior photothermal properties are synthesized *via* a hydrothermal method. These BMNS are then conjugated onto self-assembled PNFs through electrostatic interactions as well as the interactions between metal and phenolic hydroxyl groups, forming a nanohybrid material, BMNS–PNFs. The PNFs not only improve the dispersion and stability of BMNS in solution, but also enhance their biocompatibility. Subsequent photothermal performance tests of the BMNS–PNFs reveal enhanced PTT efficacy compared to BMNS alone. Furthermore, the *in vitro* cytotoxicity and cancer cell PTT efficacy tests of BMNS–PNFs demonstrate satisfied results. By introducing the concept of Ranking of Efficiency Performance (REP) method to evaluate the integrated photothermal performance of BMNS–PNFs, the excellent PTT capability of the material is demonstrated. BMNS–PNFs provide valuable inspiration for metal-based PAs based on biomaterial modification.

## 2. Materials and methods

### 2.1 Materials and reagents

The peptide with sequence KIIIIKYWYAF (purity ≥95%) was purchased from the GenScript Biotechnology Co. Ltd (Nanjing, China). NaOH (analytically pure), methylene blue (MB), CoCl_2_·6H_2_O (analytically pure) and CuCl_2_·2H_2_O were purchased from the Sinopharm Chemical Reagent Co (Beijing, China). Primary human AECII cells were obtained from the Procell Co. Ltd (Wuhan, China) and cultured in DMEM/F12 medium (Gibco, USA) with 10% fetal bovine serum (FBS) and 1% penicillin/streptomycin. Lung cancer cell lines (PC9 and HCC2279) were acquired from the Cell Bank of Type Culture Collection at the Chinese Academy of Sciences (Shanghai, China) and cultured in RPMI-1640 medium (Gibco, USA) with 10% FBS and 1% penicillin/streptomycin. All cells were cultured in a humidified environment with 5% CO_2_ at 37 °C. The cell viability was assessed using the cell counting kit-8 (CCK-8) assay (Beyotime Biotechnology, China) and calcein-AM/propidium iodide (PI) staining (Beyotime, China).

### 2.2 Self-assembly of peptides into PNFs

To prepare the peptide incubation solution, 5 mg of peptide powder was dissolved into 5 mL of deionized water (DIW). The solution was sonicated for 20 min using an ultrasonic bath to ensure complete dissolution, achieving a concentration of 1 mg mL^−1^. The peptide solution was then incubated in a water-bath at 37 °C for 5 days. During this period, small aliquots of the solution were taken daily to prepare the samples for atomic force microscopy (AFM) analysis. This allows for the observation and documentation of the peptide self-assembly process over time.

### 2.3 Synthesis of BMNS

The synthesis of BMNS was carried out with slight modification according to previous literature.^37^ In brief, 4.4 g of NaOH was dissolved into 20 mL of DIW to prepare an alkaline reaction solution. Then, 71.4 mg (15 mmol) of CoCl_2_·6H_2_O and 51.15 mg (15 mmol) of CuCl_2_·H_2_O were added into the solution. The mixture was then stirred thoroughly for 10 min and subjected to ultrasonication to ensure complete dispersion of the particles. The reaction mixture was then placed in a water-bath at 80 °C for 24 h. During this process, the solution gradually changed from blue to black, indicating the formation of BMNS. The resulting BMNS were purified by the centrifugation at 8000 rpm for 10 min, followed by washing with DIW until the pH was approximately 7. The BMNS precipitate was then freeze-dried for 24 h and stored in a dry environment at room temperature for further use.

### 2.4 Synthesis of BMNS–PNFs

The synthesized BMNS were dispersed and combined with a PNF solution that had been incubated for 5 days, maintaining a mass ratio of 1 : 1. The final concentration of the solution was adjusted to 1 mg mL^−1^. This mixed solution was then stirred at room temperature overnight to ensure thorough conjugation of BMNS onto the PNFs, resulting in the formation of BMNS–PNFs nanohybrid material.

### 2.5 Photothermal characterizations of BMNS–PNFs

Various concentrations of BMNS–PNFs were placed in 1 × 1 cm^2^ quartz cuvettes. These samples were then irradiated with an 808 nm laser at varying the power level, positioned 1 cm above the material, for 10 min. An infrared thermal imaging camera was used to capture the temperature images at different time intervals, allowing for the measurement and recording of the temperature change curve of the BMNS–PNFs solution over time. Additionally, for comparative analysis, the photothermal curves of BMNS, BMNS–PNFs, and H_2_O were measured under the same solution concentration and laser power.

### 2.6 Photothermal stability of BMNS–PNFs

To assess the photothermal stability of BMNS–PNFs, 1 mL of the nanohybrid at a concentration of 300 μg mL^−1^ was placed into a 1 × 1 cm^2^ quartz cuvette. The sample was then irradiated with an 808 nm laser at a power density of 2.5 W cm^−2^ for 10 min, followed by a cooling period with the laser turned off for another 10 min. This process constituted one cycle. The procedure was repeated for a total of four cycles, during which the photothermal response curve of the BMNS–PNFs was measured to evaluate the stability under repeated heating and cooling conditions.

### 2.7 PCE of BMNS–PNFs

The calculation of PCE (*η*) is based on previous literature.^21^ The specific calculation formula is as follows:
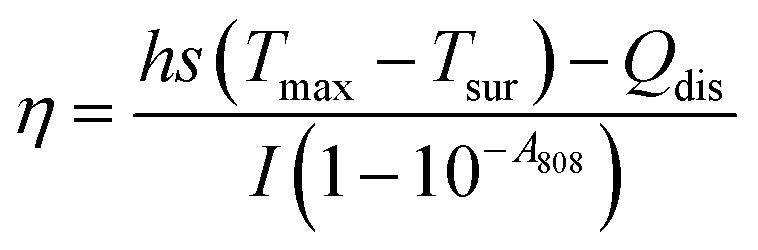
Here, *h* represents the heat transfer coefficient of the system, and *s* denotes the inner bottom surface area of the quartz cuvette (1 cm^2^). *T*_max_ is the maximum temperature achieved by the 1 mL BMNS–PNFs solution. *T*_sur_ is the ambient temperature during the measurement. *Q*_dis_ represents the heat loss during the cooling process, which can be derived from the photothermal process of the pure solvent. *I* stands for the power density of the 808 nm laser during measurement (W cm^−2^), and *A*_808_ represents the absorbance of BMNS–PNFs at 808 nm. The *hs* of the product can be obtained using the following formula:
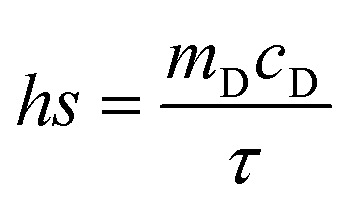
*m*_D_ and *c*_D_ represent the mass of the sample (1 g) and its specific heat capacity (4.2 J g^−1^ K^−1^), respectively. The relevant time constant (*τ*) during the cooling process is obtained using the following formula:
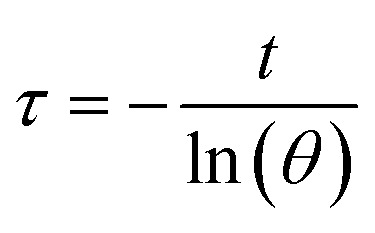

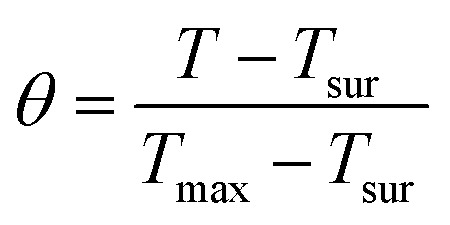
*t* represents the cooling time of the BMNS–PNFs solution. *θ* is a dimensionless constant. The heat loss during the cooling process, *Q*_dis_, can be calculated using the following formula:
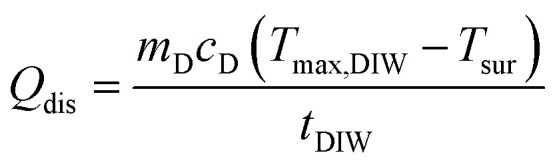
*m*_D_, *c*_D_, *T*_max,DIW_, and *t*_DIW_ represent the mass of DIW (1 g), its specific heat capacity (4.2 J g^−1^ K^−1^), the maximum temperature, and the cooling time, respectively.

### 2.8 Biocompatibility evaluation of BMNS–PNFs

Human AECII cells were selected for the evaluation of the biocompatibility of materials. The cell viability was determined using the CCK-8 assay and calcein-AM/PI staining, as described in our previous work.^5^ In summary, for the CCK-8 assay, 5000 AECII cells were seeded into 96-well plates and cultured overnight. Subsequently, 100 μL of PNFs (300 μg mL^−1^), BMNS (300 μg mL^−1^), or BMNS–PNFs (300 μg mL^−1^) were added into the culture medium, with 100 μL of culture medium serving as the control. After incubation for 0, 1, and 2 days, the medium was rinsed with PBS solution, and 100 μL of complete medium containing 10 μL of CCK-8 solution was added into each well. The optical density (OD) was measured at 450 nm using a microplate reader after 3 h of incubation. The difference in the OD values indicated the survival and proliferation of the cells. For the calcein-AM/PI staining, 5000 AECII cells were seeded into 96-well plates and cultured overnight. Then, 100 μL of culture medium containing 300 μg mL^−1^ BMNS or BMNS–PNFs was added into the wells, with 100 μL of culture medium serving as the control. After 24 h of incubation, the calcein-AM/PI solution was added into the wells and incubated for 30 min before the observation under a fluorescence microscope.

### 2.9 *In vitro* antitumor assessment of BMNS–PNFs

Two lung cancer cell lines, PC9 and HCC2279, were selected for the *in vitro* PTT evaluation. In summary, 10 000 PC9 and HCC2279 cells were seeded into each well of 24-well plates and cultured overnight. The experiment included a control group (without treatment), a laser-only group, and a group treated with BMNS–PNFs combined with laser irradiation. The laser power was set to 2 W cm^−2^ for 3 min. Following the treatment, the PC9 and HCC2279 cells were incubated for an additional 2 h. Each group of cells was then stained using the BMNS–PNFs calcein-AM/PI kit for 30 min, following the manufacturer's instructions. During staining, live cells were marked with calcein (green), while dead cells were marked with PI (red). The stained cells were subsequently examined under an inverted fluorescence microscope. Furthermore, the impact of BMNS–PNFs function on mitochondria function of HCC2279 cells were assessed by Mitochondrial Membrane Potential Assay Kit (with JC-1) (Elabscience, China) according to the manufacture's instruction. Briefly, HCC2279 cells were seeded in 24-well plates at a density of 20 000 cells per well. In addition to the control group, the cells were treated with BMNS–PNFs alone or BMNS–PNFs combined with laser irradiation. After the treatment, cells were cultured with another 4 h and then mitochondrial membrane potential were evaluated. To assess the apoptosis, HCC2279 cells were treated under specified experimental conditions and subsequently analyzed using flow cytometry. Apoptotic cells were stained with the Annexin V-FITC Apoptosis Detection Kit (Vazyme, China), following the manufacturer's protocol and as described in a previous study.^38^

### 2.10 Characterization techniques

AFM images were acquired in tapping mode using an FM-Nanoview 6800 AFM (Suzhou FSM Precision Instrument Co., Ltd, PR China). The samples consisted of 10 μL of a 0.01 mg mL^−1^ solution, which were dried on a smooth mica substrate. The Gwyddion software (Version 2.62) was utilized to generate both plane and height images. Transmission Electron Microscopy (TEM) images were obtained by depositing 10 μL of a 0.01 mg mL^−1^ sample onto a standard carbon film and capturing images with a Tecnai G2 F20 TEM (FEI Co). X-ray Photoelectron Spectroscopy (XPS) was conducted using a Thermo Scientific K-Alpha spectrometer (USA) to characterize the binding energy spectra of the materials. Ultraviolet-visible (UV-Vis) absorbance spectra were measured using a UV-1900i spectrophotometer (SHIMADZU Company, Japan). The polydispersity index (PDI), zeta potential in liquid media were investigated using dynamic light scattering (DLS) and zeta potential measurements (Zetasizer Nano ZSE, Malvern, UK). An 808 nm laser, controlled by a Laser Diode Controller (ADR-1860, PR China), was used to irradiate the BMNS–PNFs solution. Real-time temperature images and photothermal heating curves were captured and recorded using an infrared thermal imaging camera (Fotric 223 s, PR China).

## 3. Results and discussion

### 3.1 Regulation of the self-assembly of PNFs

The self-assembly behavior of peptides is primarily influenced by internal factors,^31^ such as the type, number, and arrangement of amino acids that compose the peptide, as well as external factors, including the self-assembly time, temperature, peptide concentration, and solution pH. By manipulating these internal and external factors, it is possible to control the morphology of the nanostructures formed by peptide self-assembly. For internal factors, we selected a typical self-assembling sequence of PNFs, KIIIIKYWYAF. This sequence consists of two main components, including KIIIIK, which drives the self-assembly process, and YWYAF, which allows for multifunctional modifications. Previous study has demonstrated that the amphiphilic hexapeptide KIIIIK can form fibrous structures through β-sheet folding, acting as the primary driving force for the peptide self-assembly.^36^ The YWYAF segment can adjust the overall isoelectric point of the peptide, thereby modulating the charge of PNFs in aqueous solution, which facilitates the subsequent loading of BMNS onto PNFs.

**Fig. 1:**
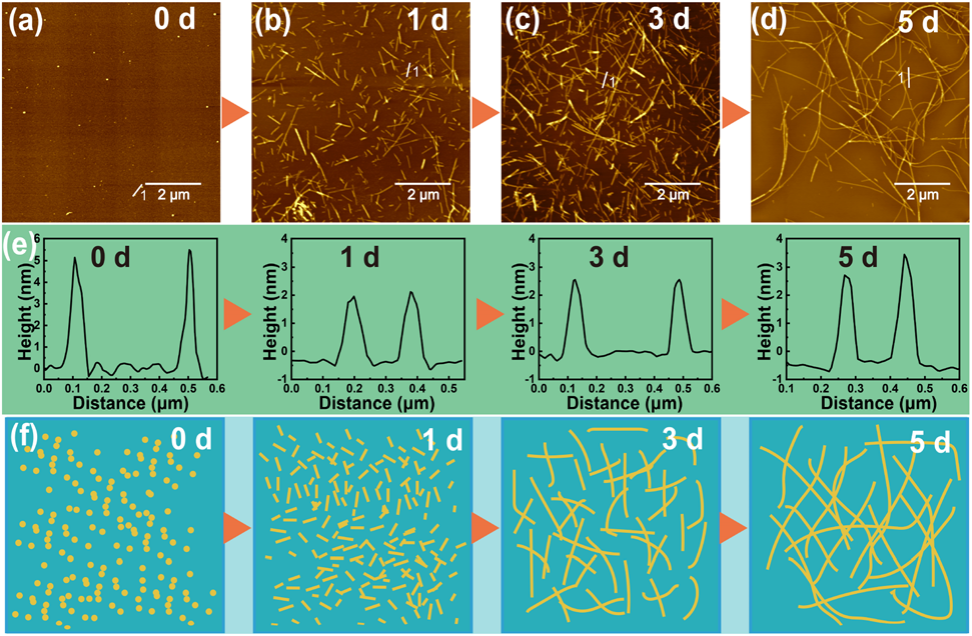
Schematic illustration of the self-assembly of PNFs: (a) day 0. (b) day 1. (c) day 3. (d) day 5. (e) Plot of cross-section analysis of PNFs at different time periods. (f) Diagram of the potential self-assembly process.

For the regulation of external factors, DIW was selected as the solvent, and a peptide concentration of 1 mg mL^−1^ was used for self-assembly at 37 °C. As shown in Fig. 1a–f, peptide molecules exhibit different aggregation states over time, with a trend of increasing PNF length. Initially, when the peptide molecules are dispersed in DIW (Fig. 1a), they form particles with a diameter of approximately 5 nm due to incomplete dispersion. However, this state is unstable, and the nanoparticles further disperse into individual peptide molecules. Due to non-covalent interactions, after one day of incubation, the dispersed peptide molecules in DIW begin to aggregate in an orderly manner, forming shorter PNFs (Fig. 1b). These PNFs are measured to be 1–2 μm in length and 2–4 nm in diameter, laying the groundwork for subsequent growth. After three days of incubation (Fig. 1c), the length of PNFs generally exceeds 5 μm, with little change in diameter. As the incubation time increases, the length of PNFs continues to grow. By the fifth day of incubation (Fig. 1d), PNFs exceed 10 μm in length, demonstrating a well-defined self-assembled morphology. The cross-sectional analysis of the formed PNFs shows that the PNFs have a diameter of 2–4 nm and did not change with time (Fig. 1e). Additionally, PNFs in aqueous solution exhibit excellent stability, maintaining their fibrous state even after one month at room temperature. Aqueous solutions of PNFs with colloidal stability address the stability of metallic nanomaterials in aqueous media.^39^Fig. 1f illustrates the potential self-assembling process of peptide molecules into PNFs over time: initially forming shorter PNFs in solution, which then continue to grow into longer PNFs. PNFs provide an excellent building block for BMNS and enhance the stability of nanohybrid materials in aqueous solutions.

### 3.2 Synthesis and characterizations of BMNS–PNFs hybrids

**Fig. 2:**
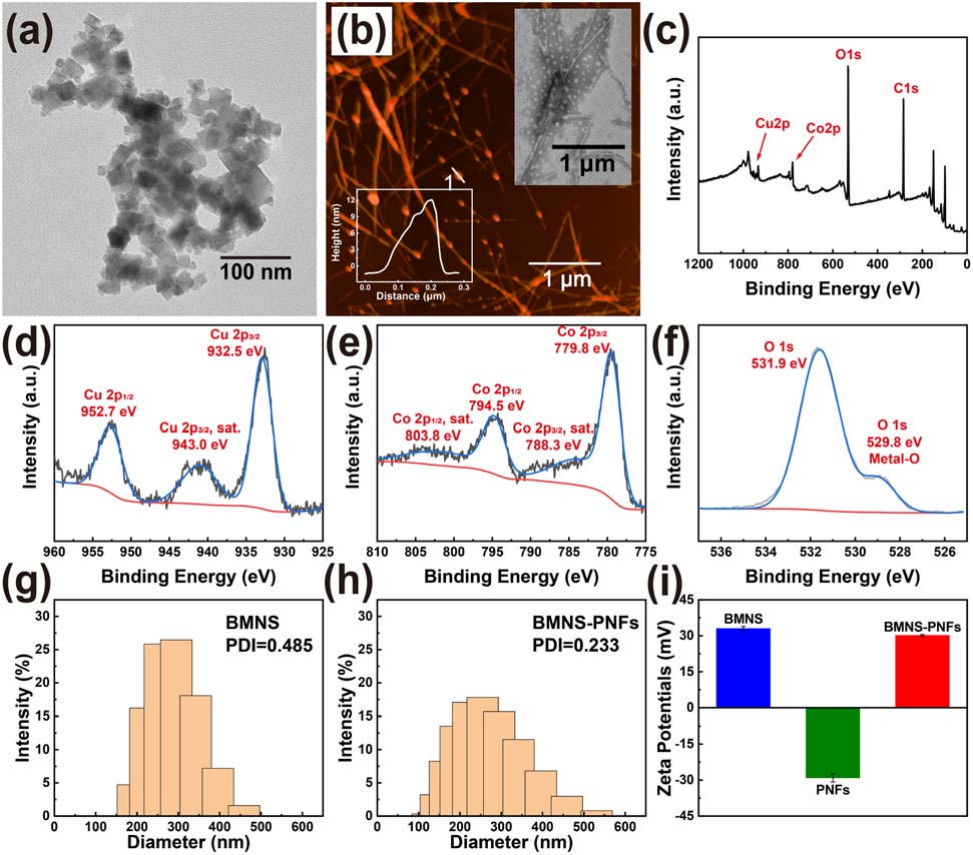
Morphology, spectra and DLS characterization of BMNS–PNFs: (a) TEM image of BMNS. (b) AFM and TEM images and height analysis plots of BMNS–PNFs. (c) Overall XPS spectrum. (d–f) Electron binding energy analysis of the Cu, Co, and O elements. (g and h) Hydrodynamic sizing and PDI analysis of BMNS and BMNS–PNFs. (i) Zeta potential analysis of BMNFs, PNFs and BMNS–PNFs.

The size and shape of nanomaterials significantly influence their light absorption and scattering properties, thereby affecting their photothermal effects.^40^ Firstly, the morphology of the synthesised BMNS and BMNS–PNFs were characterized correlatively. The synthesized CuCoO_2_ nanosheets are approximately 10 nm in size (Fig. 2a) and exhibit a layered structure. Compared to spherical nanoparticles, these sheet-like BMNS can generate localized electromagnetic field enhancements, leading to improved PCE. To further enhance the biocompatibility and photothermal conversion properties of BMNS, we selected PNFs that incubated for 5 days as carriers to regulate the arrangement of BMNS. By adjusting the PNF solution to a mildly alkaline condition (pH = 8), the phenolic hydroxyl groups on the PNFs lose hydrogen ions, resulting in negative charges on the PNFs. Meanwhile, the Cu and Co metals within the BMNS exhibit a positively charged state in aqueous solutions. Consequently, BMNS can be loaded onto PNFs through electrostatic and metal–phenolic hydroxyl interactions, displaying a linear arrangement (Fig. 2b).

Secondly, to determine the elemental composition and the electronic states of elements within the synthesized BMNS–PNFs, XPS analysis was conducted on the BMNS–PNFs composites (Fig. 2c–f). The peaks at 952.7 and 932.5 eV are attributed to the binding energies of Cu^+^ 2p_1/2_ and Cu^+^ 2p_3/2_, respectively (Fig. 2d). In addition, the peaks at 794.5 and 779.8 eV, along with two shake-up satellite peaks at 803.8 and 788.3 eV (marked as “sat.”), correspond to the binding energies of Co^3+^ 2p_1/2_ and Co^3+^ 2p_3/2_, respectively (Fig. 2e). The peak at 531.9 eV corresponds to the binding energy of O 1s, while the smaller peak at 529.8 eV is associated with the binding energy of hydroxyl oxygen bound to metal (Fig. 2f). Therefore, the XPS characterization demonstrates the successful synthesis of BMNS and its effective loading onto PNFs.

Finally, DLS tests were performed on BMNS and BMNS–PNFs. Among them, the hydrodynamic diameter of BMNS is in the range of 150–500 nm and its PDI value is 0.485 (Fig. 2g). In comparison, the hydrodynamic diameter of the hybridized material BMNS–PNFs is in the range of 100–600 nm, and its PDI value is 0.233 (Fig. 2h). Therefore, it is not difficult to find that the hydrodynamic diameter increases after hybridization, which is due to the fact that the BMNS aggregates on the PNFs thus increasing the measured particle size, which also confirms the interaction between the BMNS and the PNFs side by side. In addition, according to previous studies,^41^ a PDI value equal to or lower than 0.3 indicates an acceptable uniform particle size distribution and stability. Therefore, we suggest that BMNS–PNFs have better stability and homogeneity compared to BMNS, which is significant for storage and biodistribution. Meanwhile, in order to verify the electrostatic interactions between BMNS and PNFs, we did zeta-potential tests (Fig. 2i) on BMNS, PNFs, and BMNS–PNFs, and the results showed that there were electrostatic interactions between BMNS and PNFs and that all the three substances had better stability.

### 3.3 Photothermal effects of BMNS–PNFs nanohybrids

**Fig. 3:**
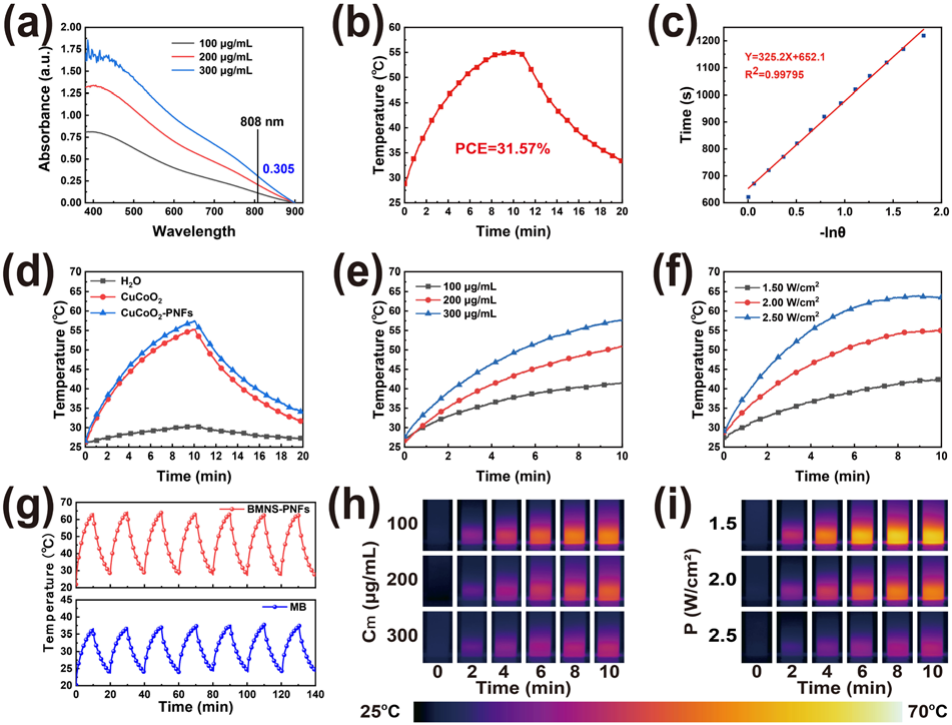
Photothermal properties of BMNS–PNFs: (a) UV analysis. (b–d) PCE testing of BMNS–PNFs. (e and h) Photothermal heating curves and real-time temperature images of BMNS–PNFs at different concentrations under a power density of 2.00 W cm^−2^. (f and i) Heating curves and real-time temperature images of 300 μg mL^−1^ BMNS–PNFs under different power densities. (g) Photothermal stability tests of BMNS–PNFs and MB.

As a type of plasmonic nanomaterial, BMNS–PNFs can absorb NIR light at specific angles and wavelengths, generating high-energy hot electrons.^42^ These hot electrons can convert light energy into thermal energy through the electron–phonon relaxation process, releasing heat to the surrounding liquid. This process constitutes the photothermal conversion of BMNS–PNFs. To evaluate the PCE of BMNS–PNFs, the UV testing was performed first (Fig. 3a). The UV absorbance of BMNS–PNFs is concentration-dependent, with a recorded absorbance of 0.305 at 808 nm for 300 μg mL^−1^ BMNS–PNFs. Subsequently, the temperature–time curves for heating and cooling, each lasting 10 min, were measured for 300 μg mL^−1^ BMNS–PNFs under a power density of 2 W cm^−2^ (Fig. 3b). A plot of time *versus* −ln(*θ*) during the cooling phase was fitted (Fig. 3c), yielding a time constant of 326.2. Based on this, the PCE of BMNS–PNFs was calculated to be 31.57%.

In addition, the photothermal heating curves of three materials, including H_2_O, BMNS, and BMNS–PNFs, were tested (Fig. 3d). It is evident that both BMNS and BMNS–PNFs exhibit excellent photothermal effects compared to pure water. Notably, the photothermal performance of the BMNS–PNFs nanohybrid material is slightly superior to that of BMNS, suggesting that PNFs enhance the photothermal properties of BMNS due to synergistic effect.

To further investigate the relationship between the photothermal effect of BMNS–PNFs and its concentration as well as the power density of an 808 nm laser, we examined the photothermal heating curves of BMNS–PNFs at various material concentrations under a power density of 2.00 W cm^−2^ (Fig. 3e) and captured real-time temperature images (Fig. 3h). Additionally, we assessed the heating curves (Fig. 3f) and real-time temperature images (Fig. 3i) of BMNS–PNFs at a concentration of 300 μg mL^−1^ under different power densities. These experiments demonstrate that the heating rate of BMNS–PNFs nanohybrid materials is dependent not only on the material concentration, but also on the power density. Meanwhile, a concentration of 300 μg mL^−1^ of BMNS–PNFs and a power density of 2.00 W cm^−2^ were chosen as the benchmarks for subsequent experiments.

Finally, the photothermal stability of BMNS–PNFs and MB was tested in order to demonstrate the excellent photothermal stability of BMNS–PNFs (Fig. 3g). 300 μg mL^−1^ of BMNS–PNFs and MB were selected for seven consecutive warming–cooling cycles at a power density of 2.5 W cm^−2^. MB, as a photosensitiser with better photothermal stability, can be used here as a comparison of the photothermal stability of BMNS–PNFs.^43^ After several heating–cooling cycles, BMNS–PNFs still had excellent photothermal effects, proving its excellent photothermal stability. In addition, the photothermal stability of BMNS–PNFs was comparable to that of MB, and the photothermal effect of BMNS–PNFs at the same concentration was more obvious. Therefore, BMNS–PNFs nanohybrid materials can be an option for PTT.

### 3.4 Biocompatibility evaluation of BMNS–PNFs

**Fig. 4:**
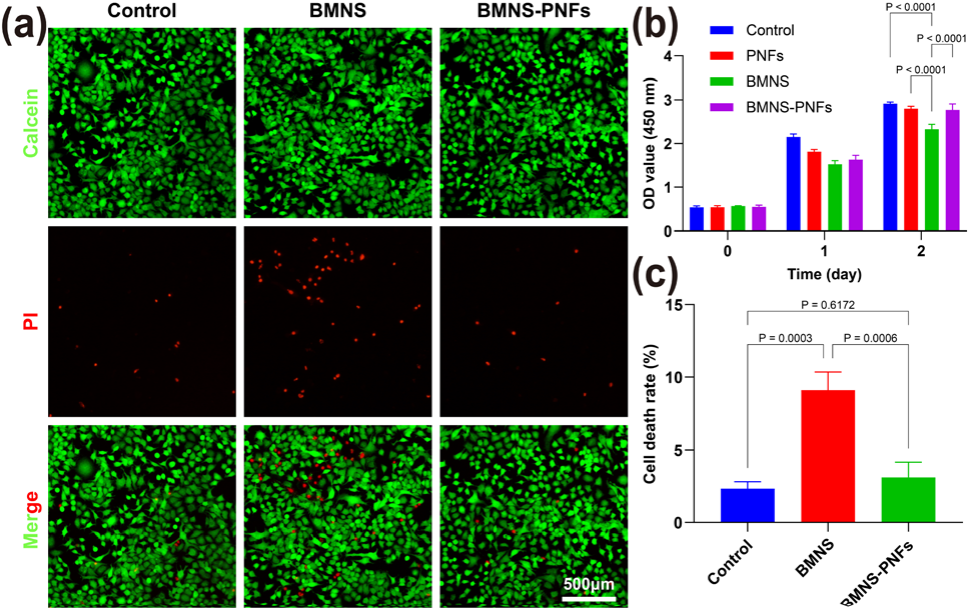
Cytotoxicity of BMNS–PNFs on human AECII cells. (a) Fluorescence microscope images of human AECII cells stained by calcein AM and PI. (Scale bar = 500 μm). (b) CCK-8 results of different treatments on human AECII cells. (c) Statistical analysis of calcein AM/PI staining.

Biocompatibility is a vital factor in designing biomaterials for anticancer therapies. It ensures that these materials effectively target and eliminate tumor cells without damaging healthy cells, which is crucial for their eventual clinical use.^44^ We evaluated the cytotoxicity of BMNS–PNF at a concentration of 300 μg mL^−1^ on human AECII cells using the CCK-8 and calcein-AM/PI staining methods. As the calcein-AM/PI staining illustrated in Fig. 4a and c, the treatment with BMNS alone decreased the viability of human AECII cells, suggesting potential toxicity to normal cells. However, the treatment with BMNS–PNFs did not show any significant harm to human AECII cells. As illustrated in Fig. 4b, the CCK-8 results indicate that PNFs alone did not display toxicity to human AECII cells, and their conjugation with BMNS reduced the toxicity of BMNS on normal cells after two days of co-incubation. The results are consistent with our previous findings that the PNFs could enhance the biocompatibility of biomaterials on cells.^39^ Overall, we suggest that the formed BMNS–PNFs demonstrated good biocompatibility with normal cells, indicating their potential for further use in anticancer treatments.

### 3.5 *In vitro* antitumor assessment of BMNS–PNFs

**Fig. 5:**
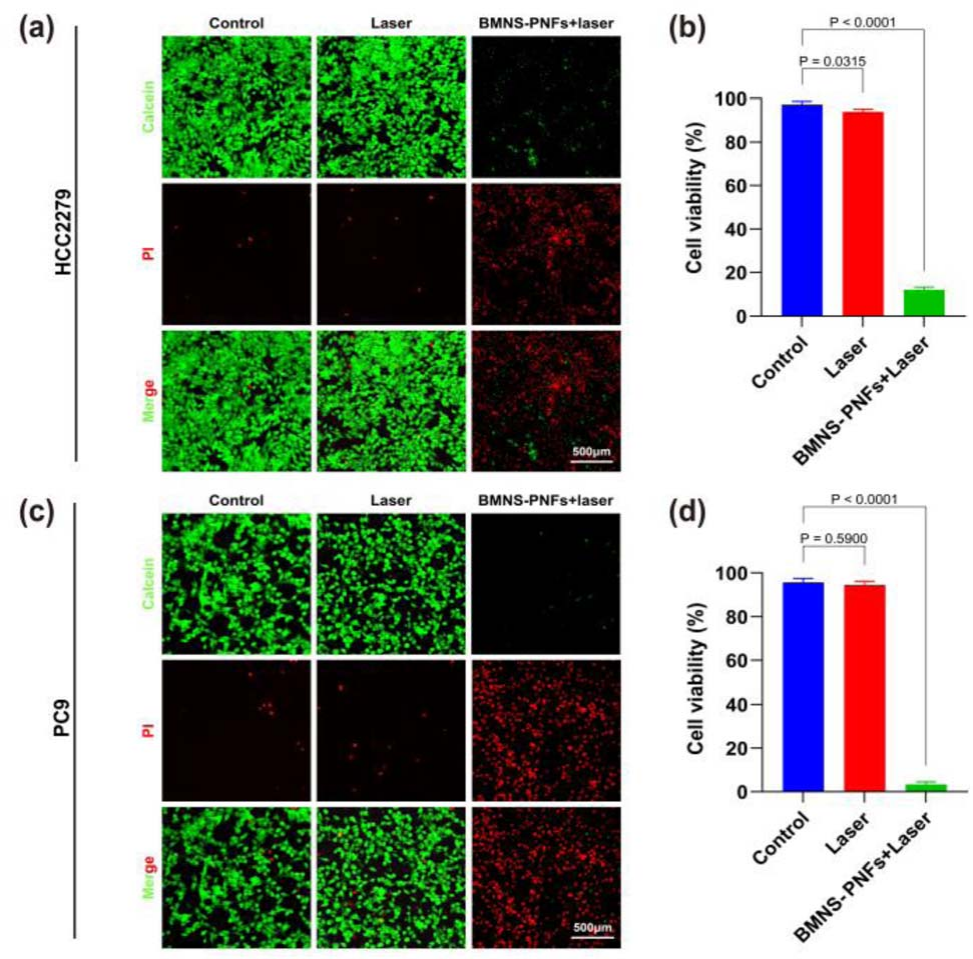
Antitumor effect of BMNS–PNFs on HCC2279 and PC9 lung cancer cells: (a) fluorescence microscope images of HCC2279 cells stained by calcein AM and PI with different treatments. (b) Statistical analysis of calcein AM/PI staining for HCC2279 cells. (c) Fluorescence microscope images of PC9 cells stained by calcein AM and PI with different treatments. (d) Statistical analysis of calcein AM/PI staining for PC9 cells.

We then assessed the antitumor effects *in vitro* using lung cancer cells (HCC2279 and PC9) as the target cells. As shown in Fig. 5a and c, the control group treated with saline displayed minimal red fluorescence, indicating rapid proliferation of tumor cells. After the irradiation with a 2 W cm^−2^, 808 nm laser for 3 minutes, the red fluorescence in the laser-only treatment group was similar to that of the control group, suggesting that the 808 nm laser alone was ineffective in killing HCC2279 or PC9 cancer cells.

In contrast, the BMNS–PNFs at a concentration of 300 μg mL^−1^ group exhibited strong red fluorescence across almost the entire visual field, with minimal green fluorescence, indicating a cell death rate of over 90% for HCC2279 cells and over 95% for PC9 cells which is shown in Fig. 5b and d. These results demonstrate that the PTT using BMNS–PNFs can effectively eradicate cancer cells, highlighting BMNS–PNFs as a promising material for the tumor treatment.

**Fig. 6:**
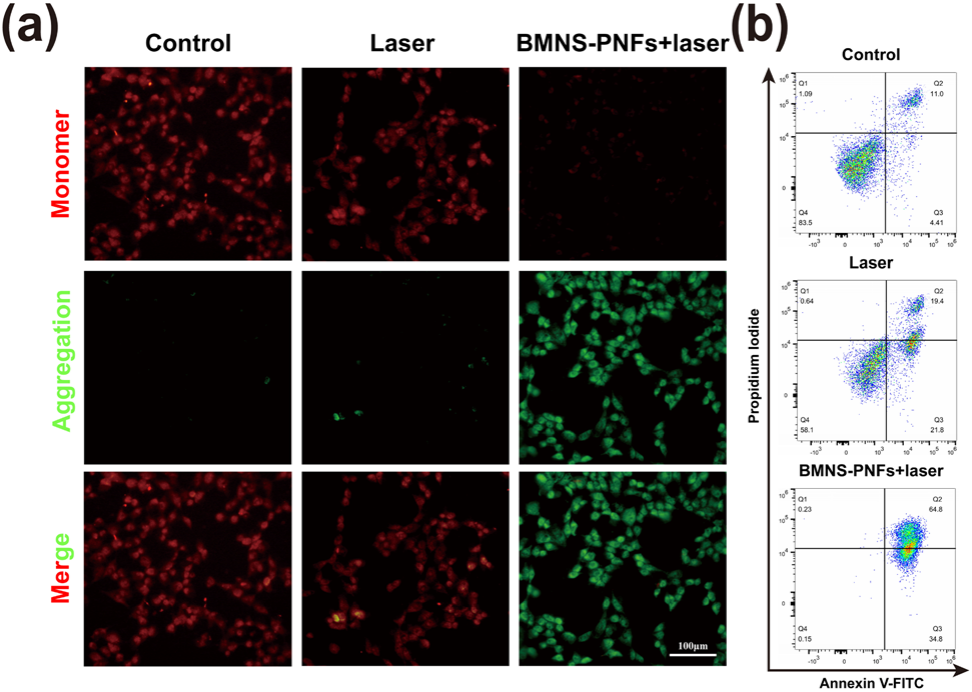
Antitumor mechanism of PTT on HCC2279 cells *via* mitochondrial targeting: (a) fluorescence microscope images of HCC2279 cells stained with JC-1 probes, showing mitochondrial membrane potential under different treatment conditions. (b) Apoptosis analysis of HCC2279 cells under different treatments, assessed through Annexin V-FITC/PI double staining and flow cytometry. Scale bar = 100 μm.

Previous studies have demonstrated that mitochondria are highly sensitive to heat, making them an excellent target for organelle-specific PTT.^45,46^ The mitochondrial membrane potential (MMP) serves as a critical indicator of mitochondrial damage. To assess this, we analyzed the MMP of HCC2279 cells under different treatments using JC-1 staining. As shown in Fig. 6a, the HCC2279 cells treated with BMNS–PNFs combined with laser irradiation exhibited the strongest green fluorescence intensity, indicating a significant decline in MMP. This suggests that BMNS–PNFs combined with laser treatment induced severe mitochondrial dysfunction. We further evaluated the apoptosis ratio of HCC2279 cells under different treatments using flow cytometry. As shown in Fig. 6b, the apoptosis ratio of HCC2279 cells treated with BMNS–PNFs combined with laser irradiation reached 99.6%, significantly higher than in other groups. These findings demonstrate the excellent antitumor effects of BMNS–PNFs under laser irradiation, likely attributed to mitochondrial damage as the underlying mechanism.

Cells, as complex fundamental units of life, exhibit varied responses to stimuli at different temperatures, which in turn influences the mechanism of the PTT process at the cellular level.^47^ The effectiveness of PTT critically depends on maintaining a reasonable temperature range. Excessively high temperatures can damage normal tissue cells, while temperatures that are too low may not effectively kill cancer cells.^48^

To achieve optimal therapeutic effects in PTT, the temperature is typically maintained between 37–55 °C, as cancer cells exhibit different states at various temperatures. When the temperature surrounding cancer cells is slightly above normal body temperature (≤41 °C), cellular homeostasis experiences fluctuations, but this process remains reversible, and the rate of cellular transmembrane diffusion increases. As the temperature rises further (41–50 °C), partial cytoskeletal damage occurs, the synthesis of heat shock proteins increases, and the pH of the cytoplasm decreases, resulting in cellular acidosis. For effective cancer cell ablation, the temperature must exceed 50 °C.

**Fig. 7:**
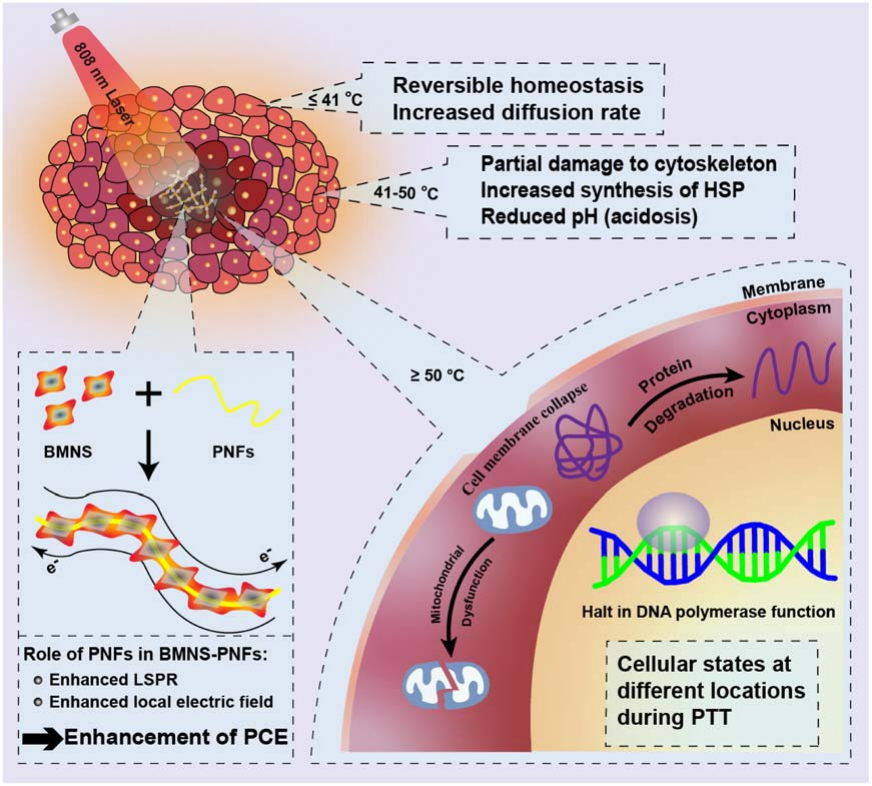
Schematic diagram of the mechanism of thermal ablation of cancer cells and the enhancement of PCE by PNFs during PTT.

At a concentration of 300 μg mL^−1^, BMNS–PNFs achieved a temperature of 55 °C within 10 min at a power density of 2 W cm^−2^, facilitating the thermal ablation of cancer cells. At this elevated temperature, mitochondrial function in cancer cells is compromised, proteins are denatured, and the activity of DNA polymerase in the nucleus ceases, thereby promoting cancer cell death. We propose a potential mechanism of BMNS–PNFs towards PTT of cancer cells, as shown in Fig. 7. As a surface plasma, BMNS can absorb light energy to generate high-energy hot electrons and enhance the electromagnetic field, which in turn releases thermal energy through the electron–phonon relaxation process, exhibiting excellent photothermal conversion capabilities.^40^ The incorporation of PNFs loaded with BMNS, which contain abundant π–π conjugation, can enhance the PCE of the nanohybrid materials. This enhancement is likely due to the π–π conjugation increasing the electron cloud density and enhancing electron mobility on the surfaces of the BMNS–PNFs. Consequently, there is an enhancement in localized surface plasmon resonance (LSPR) and the localized electric field under near-infrared light irradiation. Additionally, the higher electron cloud density provides more sources for the generation of high-energy hot electrons, increasing the thermal energy release and thus further enhancing the PCE of BMNS–PNFs.

### 3.6 REP analysis of BMNS–PNFs

**Fig. 8:**
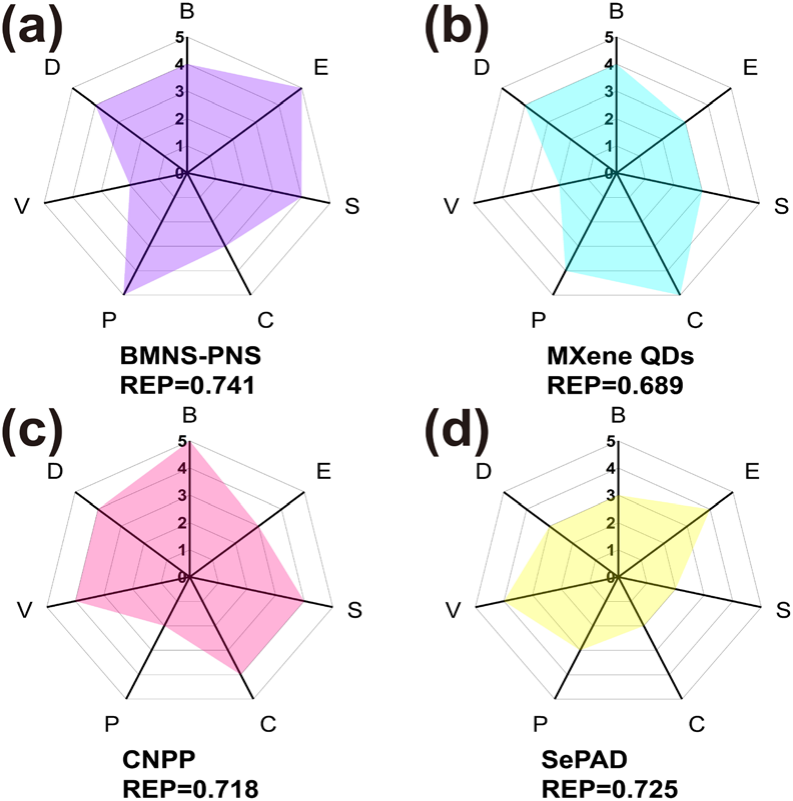
REP analysis of different PAs: (a) MXene QDs. (b) CNPP. (c) SePAD. (d) BMNS–PNFs. Various properties taken into account: biosafety (B), economy (E), stability (S), conversion efficiency (C), simplicity of preparation (P), versatility of function (V), and dispersion (D).

The comprehensive performance of PAs is crucial for tumor PTT applications and determines the suitability of PAs for the PTT process. To assess the overall capability of PAs in PTT, we employed the REP method that proposed by Mezzenga *et al.* to analyze the performance, cost, and biosafety of BMNS–PNFs. The combined efficacy of BMNS–PNFs in the PTT process was evaluated by comparing their performance with three previously reported PAs, including MXene QDs,^49^ CNPP,^50^ and SePAD,^51^ and calculating the REP of all four PAs (Fig. 8a–d).

In the analysis process, the biosafety (B) specifically refers to the biocompatibility of the PA, the economy (E) indicates whether the raw materials for synthesizing the PA are inexpensive and readily available, and the stability (S) denotes the resistance of the photothermal agent to decomposition or aggregation during photothermal conversion. The conversion efficiency (C) refers to the PA's ability to convert light into heat, the simplicity of preparation (P) pertains to the ease of the preparation process and simplicity of the required equipment, the versatility (V) indicates additional functionalities of the photothermal agent, and the dispersion (D) refers to the uniformity of dispersion of nanohybrid materials in aqueous solution.

By analyzing these six factors across the four hybrid PA materials, it becomes evident that the BMNS–PNFs nanohybrid materials exhibit certain advantages in their overall performance compared to other materials. Furthermore, the calculated REP values for MXene QDs, CNPP, SePAD, and our BMNS–PNFs are 0.689, 0.718, 0.725, and 0.741, respectively. A comprehensive assessment of the integrated properties can help to some extent in the biomedical applications of nanohybrid materials. With a sufficient number of factors assessed, the comprehensive performance of the material can be better demonstrated, facilitating the assessment of the stability of the material *in vitro*, the safety of the *in vivo* circulation, and the efficiency of the tumor treatment.^52^ Therefore, we suggest that the BMNS–PNFs prepared in this study hold significant promise for advancing the biomedical applications of tumor PTT.

## 4. Conclusion

In summary, we developed a functional nanohybrid for tumor PTT and validated its PCE, biocompatibility, and cytotoxicity against tumor cells. By utilizing the self-assembly of motif-designed peptide molecules in solution, we constructed PNFs with diameters ranging from 2 to 4 nm and lengths extending to several μm. Subsequently, we synthesized BMNS with excellent photothermal properties through a one-step hydrothermal method and successfully loaded them onto the PNFs. The resulting BMNS–PNFs demonstrated high biocompatibility and remarkable PTT efficiency, achieving a PCE of 31.57%. Furthermore, the superior performance of this material for tumor PTT was confirmed by comparing the combined performance of four different PAs across six different factors using the REP method.

This work paves the way for the development of bimetallic nanomaterials that integrate biomolecules for PTT, offering a fresh perspective on the design and advancement of photothermal biomaterials. Looking ahead, further research will concentrate on optimizing the design and synthesis of peptide nanoassemblies, as well as enhancing metal-based PAs to facilitate more effective biosafety assessments and functionalization. This will lead to the development of safer and more efficient material solutions for cancer therapy and other biomedical applications. Moreover, with the ongoing progress in nanotechnology and biomaterials science, the synergy between metal PAs and self-assembled peptide hybrid materials holds great promise. This combination is expected to enhance localized therapeutic effects while safeguarding overall patient health and safety. Ultimately, these advancements will open up new avenues for personalized medicine and precision therapy, significantly improving tumor treatment outcomes.

## Data availability

All data supporting the findings of this study are included within the manuscript.

## Author contributions

Mingjin Xu: investigation, data curation, writing – original draft. Youyin Xu: investigation, data curation, writing – original draft. Chenxi Du: data curation, software. Guanghui Gu: data curation, writing – original draft, supervision. Gang Wei: conception, supervision, funding, writing – review & editing.

## Conflicts of interest

The authors declare that there are no conflicts of interest.
